# The Role of Mock Reviewing Sessions in the National Research Mentoring Network Strategic Empowerment Tailored for Health Equity Investigators: A Randomized Controlled Study

**DOI:** 10.3390/ijerph20095738

**Published:** 2023-05-08

**Authors:** Mohamed Mubasher, Thomas Pearson, Muhammed Y. Idris, Kimberly Lawson, Jada Holmes, Priscilla Pemu, Adriana Baez, Jonathan K. Stiles, Maritza S. Salazar, Winston E. Thompson, Alexander Quarshie, Lee S. Caplan, Yulia Strekalova, Elizabeth Ofili

**Affiliations:** 1 Department of Community Health & Preventive Medicne, Microbiology, Biochemistry and Immunology, and Clinical Research Center, Morehouse School of Medicine, Atlanta, GA 30310, USA; 2 Department of Epidemiology and Health Services Research, University of Florida, Gainesville, FL 32611, USA; 3 Department of Pharmacology and Otolaryngology, School of Medicine, University of Puerto Rico, San Juan, PR 00925, USA; 4 Department of Organization and Management, University of California, Irvine, CA 92697, USA

**Keywords:** grant writing, mock study review, career advancement, early stage investigators, health equity

## Abstract

The National Research Mentoring Network (NRMN) Strategic Empowerment Tailored for Health Equity Investigators (SETH) study evaluates the value of adding Developmental Network to Coaching in the career advancement of diverse Early-Stage Investigators (ESIs). Focused NIH-formatted Mock Reviewing Sessions (MRS) prior to the submission of grants can significantly enhance the scientific merits of an ESI’s grant application. We evaluated the most prevalent design, analysis-related factors, and the likelihood of grant submissions and awards associated with going through MRS, using descriptive statistics, Chi-square, and logistic regression methods. A total of 62 out of 234 applications went through the MRS. There were 69.4% that pursued R grants, 22.6% career development (K) awards, and 8.0% other grant mechanisms. Comparing applications that underwent MRS versus those that did not (N = 172), 67.7% vs. 38.4% were submitted for funding (i.e., unadjusted difference of 29.3%; OR = 4.8, 95% CI = (2.4, 9.8), *p*-value < 0.0001). This indicates that, relative to those who did not undergo MRS, ESIs who did, were 4.8 times as likely to submit an application for funding. Also, ESIs in earlier cohorts (1–2) (a period that coincided with the pre COVID-19 era) as compared to those who were recruited at later cohorts (3–4) (i.e., during the peak of COVID-19 period) were 3.8 times as likely to submit grants (*p*-value < 0.0001). The most prevalent issues that were identified included insufficient statistical design considerations and plans (75%), conceptual framework (28.3%), specific aims (11.7%), evidence of significance (3.3%), and innovation (3.3%). MRS potentially enhances grant submissions for extramural funding and offers constructive feedback allowing for modifications that enhance the scientific merits of research grants.

## 1. Introduction

Early-Stage Investigators’ (ESIs) research skills development is a paramount mission of many academic institutions in the USA [1,2,3,4,5,6,7,8,9]. A pivotal strategy to achieve such mission is through mentoring and training [4,5,6,7,8,9]. Nevertheless, previous studies indicated that researchers from less privileged/underrepresented societies have less access to quality traditional dyadic mentoring [10,11]. Ginther et al. also reported an alarming differences in award rates for research grant (R01) applications between White and Black applicants, albeit adjusting for several confounders [10]. Recently, Ransdell et al., 2021 [1] reported results based on 46 PsychINFO, CINAHL, and PubMed published papers between 2010 and 2020, impediments to research development among ESIs from underrepresented minority faculty that included bias, discrimination, isolation, an institutional lack of mentors, and lack of appreciation of experience and/or expertise. Per the NIH, “an ESI is an investigator on a research track to becoming a Program Director or a Principal Investigator (PD/PI) after completing his/her terminal research degree or end of post-graduate clinical training (whichever date is later) within the past 10 years and who has not previously successfully competed as a PD/PI for a substantial NIH independent research award (Investigator Career Stage Benefits NIH Center for Scientific Review”. In our NRMN SETH study, ESIs are investigators on a research career track but have not yet received independent NIH R01 or equivalent funding.

Our primary hypothesis is that developmental networks (DN) when added to grant-writing coaching can significantly enhance ESIs’ research careers [4,12]. Our NIH-sponsored Seth nested cluster-randomized study [5,6,7,8,9,12] was launched to specifically determine the magnitude and significance of adding DN to structured coaching, among diverse ESIs. Notably, the ESIs’role in the study is to work (within their institutions) closely with their study-assigned coaches, mentors and, if randomized to the DN group, their network study [5,6,7,8,9,12] assigned developer, to prepare and submit, within 12 months of recruitment into the study, grant application(s) for the NIH K, R, U and/or minority supplement awards or other comparable federal funding mechanisms. 

### 1.1. Mock Review Sessions (MRS)

To address the mission of enhancing the research experience of the NRMN SETH’s ESIs, we added to our curricula of training a focused NIH-formatted MRS emphasizing NIH criteria of scientific rigor and reproducibility.

#### 1.1.1. NIH Mock Study Section [13]

Since 2014, the Institute of Clinical and Translational Sciences (ICTS) at NIH offers NIH Mock Study Section aimed at Improving K, R, and F series grant submissions. The sessions are aimed at simulating an actual NIH study section through the Research Development Program. The review sessions for clinical and translational K, R, and F series grant applications are offered twice annually, in Spring and Winter. According to NIH, the purpose of this program is to “increase the likelihood of grant funding success by providing comprehensive, study section-like feedback to applicants on their complete grant application prior to grant submission”.

#### 1.1.2. MRS at US Institutions’ Health Science and Clinical Schools vis-à-vis MRS at NRMN SETH Program

The model of MRS has been conceptually implemented at several U.S. institutions, academic medical centers, and schools of medicine to help applicants for extramural funding to enhance their applications and maximize their chances to obtain favorable reviews that may result in funding their research (Table 1). It should be emphasized that these institutions’ MRS mechanisms are aimed at servicing their own intrinsic goals that are targeted towards their ESI scholars with no emphasis on minority subgroup inclusion. Our NRMN/SETH program is nationally focused with centralized MRS services targeted towards ESIs from smaller institutions such as the Research Centers in Minority Institutions (RCMI) which serve historically underserved students and generally have limited resources to provide NIH format and quality of MRS review of RCMI applications for funding [14,15].

### 1.2. Adding Focused NIH-Formatted MRS to NRMN SETH Training Curriculum

During the conduct of the NRMN SETH program and to enhance the ESIs’ scientific research capabilities that could potentially result in successful grant submissions, we added NIH-tailored MRS scheduled prior to the submission date of the grant applications. 

The aims of this manuscript are to (1) characterize the role of the MRS in terms of structure and utilization, (2) evaluate the association between going through an MRS and submissions and awards of extramural research grants and (3) present the most prevalent design, conduct and analysis—specific issues deemed deficient by the mock reviewers.

## 2. Materials and Methods

**Figure 1:**
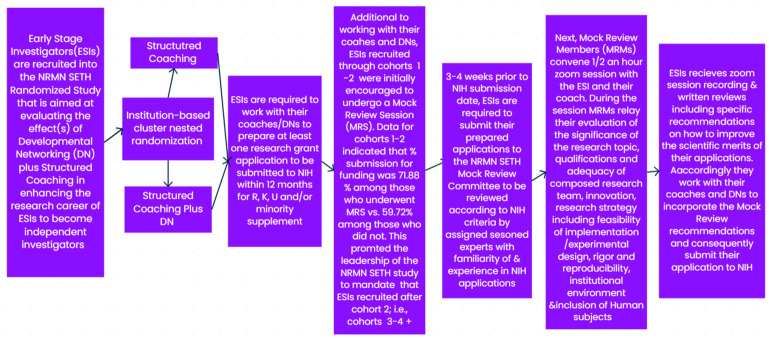
Flowchart of the interfacing of the design of the randomized NRMN SETH Study with the mock reviewing process.

Our study has thus far recruited 210 ESI participants since 14 December 2019, through four cohorts sequentially recruited over time. ESIs are required to work with their coaches and developers (for those randomized to the coaching plus DN group) and submit at least one NIH extramural application for funding within the first 12 months of their enrollment in the program. MRS were optional for cohorts 1–2 and became mandatory starting July 2020 for subsequent cohorts. Figure 1 illustrates the interfacing of the flow of the study design with the inclusion of the MRS process.

### 2.1. Composition of the MRS Reviewers and Criteria of Review

Two to three seasoned members of the NRMN SETH study team with prior NIH reviewing experience were assigned to conduct the mock reviews. Applicants were asked to submit their completed draft applications 2–3 weeks before convening the MRS. Applications were then sent to at least two reviewers, who convened a 30-min MRS session.

The MRS were tailored after the NIH format which focuses on the scientific rigor and reproducibility [22], and feasibility of conducting and successfully completing the planned research. The NIH criteria focuses on (a) significance of the proposed research topic in terms of the pre-existing gaps in the scientific, clinical and public health literature, (b) the suitability of the investigator(s) background, experience, training and research/scientific abilities to successfully conduct and achieve the proposed research aims, (c) innovation of the methodology and approaches underlying the design and conduct of the proposed research topic, (d) appropriateness and reasonability of the proposed overall approach, strategy, methodology, and analysis to accomplishing the specific aims of the project, as well as protection of human subjects, inclusion of minorities, the inclusion of biological sex as a variable, and the inclusion of children, (e) alignment of institutional environment and available resources with implementation needs of the proposed research topic, so that the successful completion of the project is highly likely and (f) justification of human subjects inclusion and plans for their protection when they participate in the proposed research. Based on these criteria, we computed the frequency distributions of the most prevalent design, conduct and analysis-related factors that were deemed deficient and/or insufficient.

### 2.2. Conduct of the MRS

After introductions and procedure review, the applicant and coaches are muted. Each reviewer states an initial 1–9 NIH-tailored score. Consequently, each of the two reviewers gives a 10–12 min detailed NIH-style review of the application stating its strengths and weaknesses, with further discussion followed by restating priority scores. If time allows, the applicant can unmute for a few questions at the end. In 1–2 days following the MRS, feedback is sent to the applicant through live MRS video recording of the session for later review, along with the reviewers’ written critiques in NIH Summary Statement format. The intent is for the applicant to use the critiques in revising the application prior to final submission to NIH. It should also be noted that the MRS members provide scientifically-based suggestions to the respective ESI to assist in formulating remedies to address the cited critiques and enhance the scientific rigor of the application.

### 2.3. Quantitative Data Analysis Plan

#### 2.3.1. Study Variables

The primary outcomes included (1) submission and (2) award for funding of an NIH or other extramural grant application. The potential predicting covariates we considered for these analyses included (1) whether the ESI’s application underwent a mock review session (Yes/no), (2) type of sought mechanism of funding, i.e., R-series research grants, career development (K series) grants and others (Support of Competitive Research (SCORE) and/or National Science Foundation grants), and (3) Cohort effect, as a continious covariate (1, 2, 3, 4) and in separate models, to potentially measure COVID-19 effects (4) Early cohorts (1–2) (Pre-COVID19 era) vs. late cohorts (3–4) (when COVID-19 pandemic peaked). MRS were optional for cohorts 1–2 and became mandatory starting July 2020 starting with cohort 3. The unit of analysis was the ESI’s prepared grant application (whether or not submitted). There were (n = 234) prepared applications, produced by (n = 210) ESIs. Several ESIs had multiple applications, either through different mechanisms or resubmissions (Table 2).

#### 2.3.2. Data Analysis

Summary of the data is presented using frequencies and percentages of grant submissions and awards vis-à-vis MRS sought mechanism of funding, recruitment cohorts, and MRS periods (before and after it became mandatory) and bar chart graphics. We initially tested separately in a univariate manner using Chi-Square or Fisher Exact tests [23] the independent association of each of the grant submissions and awards with (a) going through an MRS (yes/no), (b) sought mechanism of funding, (c) early or late cohort recruitment and (d) MRS periods. Multivariate testing was pursued using unconditional independent (as well as interaction-based models) logistic regression [24] for assessing independent associations of these factors with the likelihood of grant submission or award.

**Figure 2:**
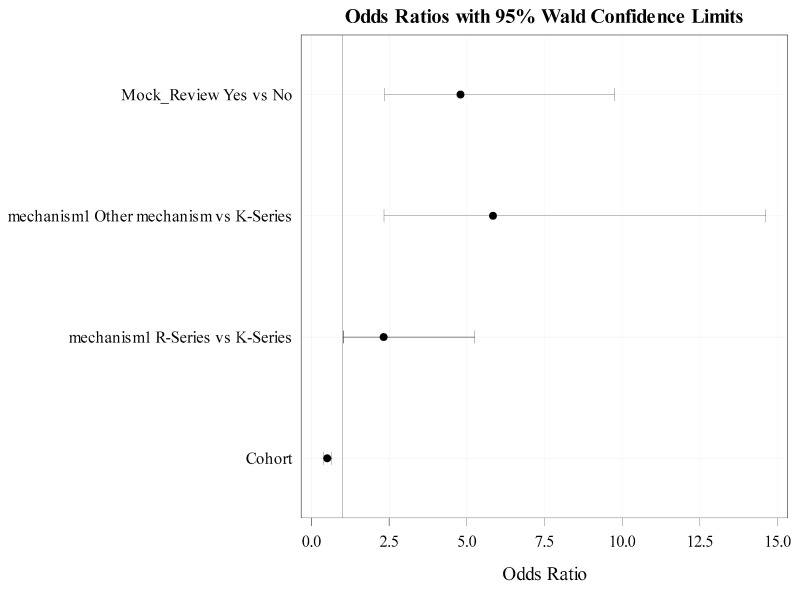
Modeling probability of applying for extramural funding with MRS period (MRS was optional for cohorts 1–2 and became mandatory for cohorts 3 and subsequent cohorts) *. * See the Methods Section for quantitative data analyses for interpretation of these graphs.

**Figure 3:**
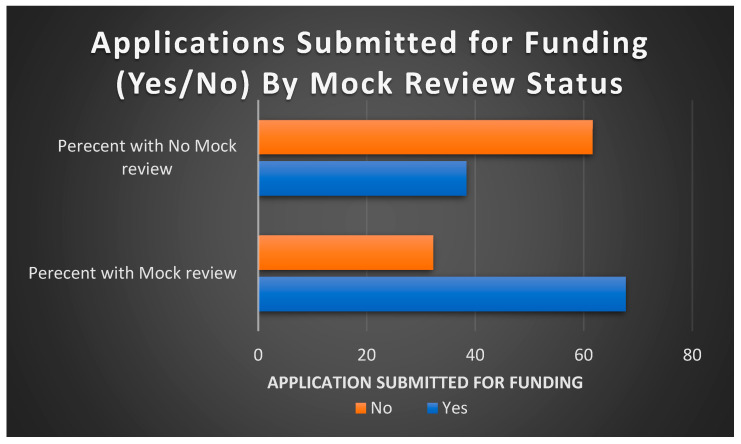
Distribution of grants submitted by mock review status.

In a mutually exclusive iterative process, we also tested cohort effects and MRS period (optional vs. mandatory). To accommodate the effects of multiple applications per ESI, additional analyses used conditional logistic regression principles to test for these effects using ESI as a cluster variable. Results are visually demonstrated using odds ratio graphs. Generally, for these graphs (c.f., Figure 2 and Figure 3 in the results section), each solid line with whiskers at the end and a dot in the middle represents the point and interval estimate (magnitude) of the Odds Ratio(s) (OR) of the outcome when comparing two subgroups of a particular independent/predictive factor (in Figure 2 for example, the dot in the middle of the top solid line represents the estimated (from the data) odds (likelihood) of applying for extramural funding for the subgroup that underwent MRS when it was optional (cohorts 1–2) divided by the corresponding odds (likelihood) of those who underwent MRS after it became mandatory (cohorts 3 + i.e., the reference subgroup). The length of the solid line in those graphs for each comparison shows the width of the 95% confidence interval of the “population” odds ratios (i.e., the range that the OR can take in the underlying population of such data). As long as the solid line does not include the value “1” (which represents equality of the two compared odds) that means the ratio of the two odds are not the same (i.e., statistically significantly different from “1” at a 0.05 significance level). The overall significance level for the test of hypotheses was set at 0.05 with provision for multiple comparisons adjustment when needed. Statistical analyses were conducted using SAS version 9.4 and R version 4.2.2

## 3. Results

Through cohorts 1–4, our study has thus far recruited 210 ESI participants that are deemed eligible to submit applications for funding. The 210 ESIs produced 234 applications for extramural funding. By October 2022, of these applications 62 (26.5%) went through an MRS with proposed mechanisms of funding distributed as follows: 43 (69.4%) R-series research grants, 14 (22.6%) career development (K series) grants and 5 (8.1%) others (Support of Competitive Research (SCORE) and National Science Foundation grants). Before and after the mock review became mandatory, 32 (51.6%) and 30 (48.4%) MRS were, respectively, held. Table 3 provides additional frequency distributions, overall, by mock review period and going through an MRS (yes/no).

### 3.1. Submission and Awards vis-à-vis MRS

Table 3 and Figure 3 indicate that when comparing applications that went through MRS (n = 62) versus those that did not (n = 172), 42 (67.7%) vs. 66 (38.4%), respectively, were submitted for extramural funding. The corresponding comparisons for applications that were awarded funding were 19.35% vs. 24.42% (8 (8.6%) of the submitted applications are still pending decision outcomes from their funding agencies/entities). Table 3 further gives frequency distributions of submitted/awarded applications by MRS period, sought mechanism of funding and recruitment cohorts.

### 3.2. Multivariate Analyses-Based Findings

**Figure 4:**
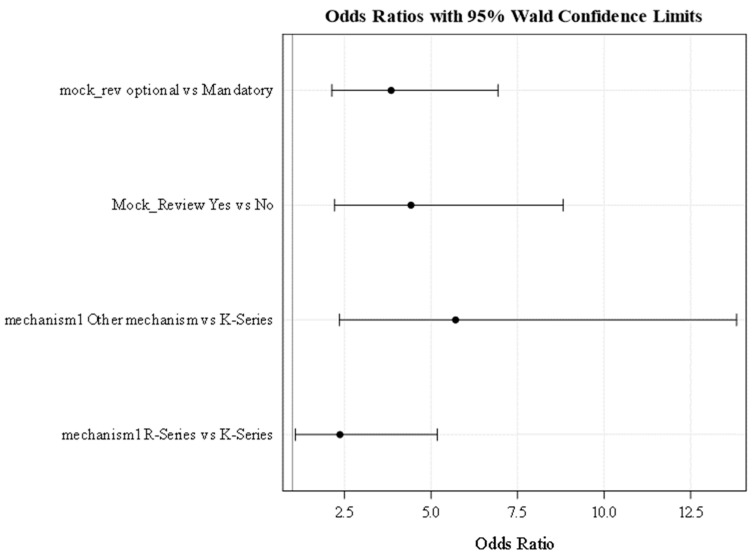
Modeling Probability of applying for NIH/Extramural Funding with cohort effect (without the covariate MRS period) *. *: See the Methods Section for quantitative data analyses for interpretation of these graphs.

Upon adjusting for sought mechanism of funding, cohort effects and (separately) MRS period, our results indicated that going through MRS (yes vs. no) was significantly associated with submitting an application for extramural funding (those who went through MRS were at least 4.8 times as likely to submit an application (OR (95% CI) and *p*-values were 4.8 (2.1, 8.7) *p*-value < 0.0001, Table 4 and Figure 4). Additionally, those who were recruited in later cohorts (cohorts 2, 3, 4 vs. cohort 1) were on average 49% less likely to submit an application for extramural funding (OR (95% CI) and *p*-value were 0.51 (0.38, 0.70) *p*-value < 0.0001 (Table 3 and Figure 3). Additionally, based on separate modeling iterations, ESIs in earlier cohorts (1–2) (a period that coincided with the pre COVID-19 era) as compared to those who were recruited at later cohorts (3–4) (i.e., during the peak of COVID-19 period), were 3.8 times as likely to submit grants (*p*-value < 0.0001) (Table 3 and Figure 2). Furthermore, as compared to those seeking K-series funding, R-series funding and other mechanisms of funding applicants were, respectively, 2.3 (*p*-value 0.042) and 5.8 (*p*-value = 0.0002) times as likely to submit for extramural funding. 

### 3.3. MRS Findings by Type of Application-Based Deficient Issues

**Figure 5:**
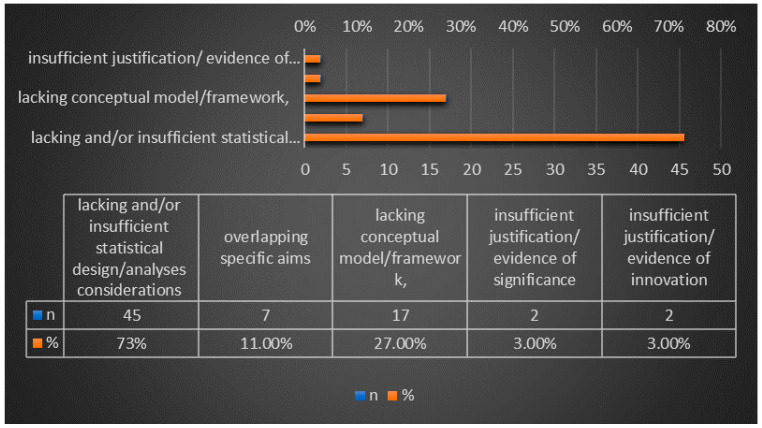
Most Prevalent deficiencies/issues (Total Number of MRS-Reviewed = 62 Applications).

The most prevalent issues (not necessarily mutually exclusive, i.e., some may overlap with each other) that were detected by the MRS were distributed as follows: 45 (75%) lacking and/or insufficient statistical design considerations and statistical analyses plan (SAP) (e.g., missing/generic sample size and statistical power justifications, and lack of clarity identifying outcome measures and covariates/confounders/predictors of outcome), 17 (28.3%) lacking conceptual model/framework, 7 (11.7%) with overlapping specific aims, 2 (3.3%) with insufficient justification/evidence of significance, and 2 (3.3%) with insufficient justification/evidence of innovation (Figure 5).

## 4. Discussion

The NRMN SETH randomized study aimed to test the effectiveness of adding DN to structured coaching in enhancing the research capabilities of ESIs to support career advancement to independent investigators.

At the beginning of this project we added NIH-type MRS to enhance the scientific rigor of ESIs’ applications for funding. 

In this manuscript, we evaluated the association between going through an MRS and the likelihood of (1) submitting an application for extramural funding and (2) being awarded funding. All submitted applications, as we speak, were included in these analyses. However, to evaluate the impact of MRS on the likelihood of being awarded funding, i.e., to properly determine the association between going through an MRS and getting funded, we needed to allow for the ”lag” time between submission and decision and consider several other predictive/mediating factors like the time-to-submission, whether for an initial application or a resubmission, the ESI’s background discipline, pre-existing self-established readiness (measured for example by number of lead-authored publications in high tier journals), existence of a mentor and existence of within-institution supporting development/incentivizing programs/resources. These factors will be considered in future analyses when we report the final findings of the NRMN SETH randomized study.

Our results showed a significant association (that can also be potentially construed as an impact) between going through an MRS and the likelihood of submitting an application for extramural funding. ESIs who went through an MRS (versus those who did not) were at least 4.8 times as likely to submit for funding (95% confidence interval = (2.8, 9.4); *p*-value < 0.0001). We intentionally focused on evaluating the potential impact of an ESI’s application going through an MRS on the likelihood of submitting an application for funding. Future analyses will investigate the association of going through an MRS vis-à-vis being awarded funding which will focus on including covariates like time-to-submission, time-to getting awarded funding and the intensity of submission/awards, i.e., multiple submissions by the same ESI.

Our analyses also revealed that earlier cohorts i.e., 1–2 versus later cohorts, i.e., 3–4, were more likely to submit an application for funding. Cohorts 1–2 were 3.8 times as likely to submit an application for funding (95% confidence interval = (2.1, 6.9); *p*-value < 0.0001). This finding was also confirmed when we portioned MRS into two periods; optional, which spanned cohorts 1–2, and mandatory, when cohorts 3–4 coincided with the MRS becoming mandatory. These findings have a very interesting implication as they point to the impact of COVID-19 on hampering ESIs’ efforts and disrupting continuity of ESIs to engage with their coaches and developers to pursue successful completion of their applications.

Multivariate analyses also indicated that R-series and other mechanisms of funding seekers (versus K-series mechanism) were, respectively, 2.3 and 5.8 times as likely to submit an application for funding (95% confidence intervals and *p*-values were, respectively, (1.03, 5.3), (2.3, 14.6) and 0.042 and 0.0002). This speaks to the composition of the ESIs in terms of their career passage and points to the fact that the risk of not submitting a K-type application is higher, likely due to the lack of strong institutional commitment, uncertain career future for postdocs, and challenges in identifying mentors with active NIH funding.

Our study also determined the most prevalent deficient issues deemed by MRS experts consequent to reviewing ESIs’ applications prior to their submission dates. Lack of or insufficient components of statistical analyses plans, like missing/generic sample size and statistical power justifications, and lack of (a) clarity identifying outcome measures and (b) covariates/confounders/predictors of outcome, were most prevalent, comprising 75% of the issues. Second to the statistical consideration issues was lack of conceptual model/framework which comprised 28.3% of the deficiencies. Other issues that were also deemed deficient were overlapping specific aims, insufficient justification/evidence of significance, and insufficient justification/evidence of innovation.

### 4.1. Strengths

To our determination, no published reports presented evaluation of MRS role vis-à-vis the likelihood of submitting for and/or being awarded extramural funding while adjusting for cohort effect and sought mechanism of funding in a structured nested cluster randomization study. The objective nature of analyses’ outcomes also bestowed rigor and reproducibility of results. Finally, analysis methodologies used for evaluating associations were statistically and mathematically sound.

The report presented here provides a rigorous base for theoretical propositions and practical suggestions. Conceptually, this study suggests that a mock study section is a unique strategy for academic development that mediates success in submitting a grant proposal. Future studies can explore the extent to which MRS promotes research self-efficacy, grant writer identity, and sense of belonging in the academic community. Practically, this study shows that since there is evidence of positive contribution of the MRS to the ESI career development progress, participation in MRS-like sessions needs to be included as a required component in grant writing programs. MRS enforces an earlier deadline for preparing a full proposal, which in turn increases the overall chances for funding for ESIs.

### 4.2. Limitations

This was a cross-sectional evaluation that did not account for the repeated nature of submitting applications for funding. Future analyses will account for repeated time-to-submission and/or getting awarded funding and the intensity of submission/awards, i.e., multiple submissions by the same ESI. Other conceivably impacting factors on the likelihood of submitting an application for funding and/or getting awarded funding, such as the ESI’s background discipline, pre-existing self-established readiness and within-institution mentoring and supporting incentivizing environments, were not included.

## 5. Conclusions

NRMN-SETH MRS offer timely, specific, and constructive feedback to ESIs to allow for potential modifications/incorporation of suggestions into their applications to help in enhancing the scientific merits of their intended research. We hope that the identified list of potential scientific deficiencies helps in articulating training curricula and protocols to enhance the scientific merits of ESIs’ applications for extramural funding. We would also like to emphasize the data-evidenced significant association and potential impact of MRS on the likelihood of pursuing extramural funding.

## Figures and Tables

**Table 1:** Sample of Mock Review Session (MRS) models at US Institutions.

Institution	MRS and Mechanism
University of North Carolina (UNC)	UNC translational and clinical sciences institute regularly offers to conduct one hour-long MRS to provide comments to the grant applications to help address potential deficiencies and to enhance their scientific merits [16].
Southern California	Southern California Clinical and Translational Science Institute also provides free MRS to (a) Increase grant-writing skills, (b) Improve scores, (c) Increase funding for USC and CHLA researchers, (d) Write effective career development and educational plans and (e) Plan strong letters of support from mentors and institutions [17].
Penn State	Penn State College of Medicine’s Mock Review of Grants (MoRe) Program offers support in advance of proposal submission. The four-stage program uses a review process similar to that of an NIH study section, but in which the reviewing team interacts with the applicant to help strengthen the proposal. The MoRe program is offered in three cycles annually, preceding each NIH grant cycle; investigators preparing non-NIH proposals are also welcome to use the program. The four stages comprise (a) preparation, (b) aims discussion, (c) specific aims revisions and (d) external review [18].
Florida State University (FSU)	Twice a year, FSU’s Office of Research Development (ORD) hosts a Mock Review Panel Program specifically for investigators working on NIH R01 proposals. Through the R01 Mock Review Panel Program, ORD offers investigators an opportunity to receive comprehensive feedback on their proposals, so that they can revise and polish their drafts and then submit to the NIH their proposals in their most persuasive form. Panelists are esteemed FSU faculty members who are selected for their past success in securing R01 grants and/or their expertise in the proposals’ unique subject areas [19].
Washington University	The Office of Training Grants (OTG) at Washington University School of Medicine in St Louis hosts NIH Training Grant (T32) Mock Study Section designed to simulate an actual NIH study section. The NIH Training grant applications submitted to the Mock Study Section are reviewed, critiqued, and scored by three faculty members and other study section members. The feedback is then distributed to applicants prior to the NIH grant deadlines [20].
Duke University	Duke University School of Nursing offers Scientific Mock Reviews facilitated by the Center for Nursing Research (CNR) to increase the likelihood of grant funding by providing comprehensive feedback to PIs and their teams. Mock sessions simulate the NIH study section review process. Applicants benefit from constructive, individualized feedback provided by a panel of their peers, and reviewers benefit by acquiring experience in the peer review process [21].

**Table 2:** Number, percentage and cumulative frequency of prepared grant applications for submission by ESIs in the NRMN-SETH Study.

No of ESIs *	No of Prepared Applications for Submission (Per ESI) *	Percent of Prepared Applications for Submission	Cumulative Frequency of Prepared Applications for Submission	Percent of ESIs	Cumulative Frequency of ESIs
188	1	81.73	188	89.52	188
20	2	8.70	228	9.52	208
2	3	1.30	234	0.95	210

*: For example, 188 ESIs prepared and submitted only one grant application; 20 ESIs prepared and submitted two grant applications and another two ESIs prepared and submitted three grant applications.

**Table 3:** NRMN SETH study frequency distributions of grant submissions and awards by mock review periods, status sought mechanism of funding and recruitment cohorts up to August, 2022.

	All		All						Mock Review Period											
									Optional (Coincided with Pre-COVID-19 era)						Mandatory (Coincided with the beginning of the Peak era of COVID-19)					
			All		Did ESI have a Mock Review Session				All		Did ESI have a Mock Review Session				All		Did ESI have a Mock Review Session			
					Yes		No				Yes		No				Yes		No	
	N	%	N	%	N	%	N	%	N	%	N	%	N	%	N	%	N	%	N	%
**Submitted Grants for Extramural Funding**																				
**Yes**	108	46.15	108	46.15	42	67.74	66	38.37	66	63.46	23	71.88	43	59.72	42	32.31	19	63.33	23	23.00
**No**	126	53.85	126	53.85	20	32.26	106	61.63	38	36.54	9	28.13	29	40.28	88	67.69	11	36.67	77	77.00
**Awarded Grants for Extramural Funding**																				
**No**	180	76.92	180	76.92	50	80.65	130	75.58	68	65.38	24	75.00	44	61.11	112	86.15	26	86.67	86	86.00
**Yes**	54	23.08	54	23.08	12	19.35	42	24.42	36	34.62	8	25.00	28	38.89	18	13.85	4	13.33	14	14.00
**Recruitment Cohort**																				
**1**	72	30.77	72	30.77	21	33.87	51	29.65	72	69.23	21	65.63	51	70.83						
**2**	31	13.25	31	13.25	11	17.74	20	11.63	31	29.81	11	34.38	20	27.78						
**3**	48	20.51	48	20.51	10	16.13	38	22.09	1	0.96			1	1.39	47	36.15	10	33.33	37	37.00
**4**	83	35.47	83	35.47	20	32.26	63	36.63							83	63.85	20	66.67	63	63.00
**Sought mechanism of funding**																				
**K-Series**	55	23.50	55	23.50	14	22.58	41	23.84	25	24.04	11	34.38	14	19.44	30	23.08	3	10.00	27	27.00
**R-Series**	116	49.57	116	49.57	43	69.35	73	42.44	49	47.12	19	59.38	30	41.67	67	51.54	24	80.00	43	43.00
**Other mechanism**	63	26.92	63	26.92	5	8.06	58	33.72	30	28.85	2	6.25	28	38.89	33	25.38	3	10.00	30	30.00

**Table 4:** NRMN SETH Study frequency distributions of grant submissions and awards by mock review periods, status sought mechanism of funding and recruitment cohorts up to August, 2022.

Effect of Predicting Covariate	OR (95% CI) *p*-Value	Interpretation
Mock Review: yes vs. no	4.8 (2.4, 9.8) <0.0001	Those who undertook MRS were 4.8 times as likely to submit a grant for extramural funding
R-series vs. K-Series	2.3 (1.03, 5.3) 0.042	R-series mechanism applicants as compared to K-series applicatnts were 2.3 times as likely to submit for a grant funding
Other-series vs. K-Series	5.8 (2.3, 14.6) 0.0002	Other-series mechanism s applicants as compared to K-series applicatnts were 5.8 times as likely to submit for a grant funding
Cohorts 1–2 vs. 3–4	3.8 (2.1, 6.9) <0.0001	ESIs in earlier cohorts (1–2) (a period that coincided with the pre COVID-19 era) as compared to those who were recruited at later cohorts (3–4) (i.e., during the peak of COVID-19 period) were 3.8 times as likely to submit grants for extramural funding (*p*-value < 0.0001)
Cohort as a continious covariate (1,2,3,4)	0.51(0.38, 0.70) <0.0001	ESIs recruited in later cohorts were on average 49% less likely to submit an application for extramural funding

## Data Availability

Data available on request due to restrictions e.g., privacy or ethical.

## List of Acronyms

NRMN	National Research Mentoring Network
ESI	Early-Stage Investigator
SETH	Strategic Empowerment Tailored for Health Equity
NIH	National Institute of Health
DN	Developmental Networking
MRS	Mock Review Session
